# Is It Possible to Predict the Odor of a Molecule on the Basis of its Structure?

**DOI:** 10.3390/ijms20123018

**Published:** 2019-06-20

**Authors:** Manon Genva, Tierry Kenne Kemene, Magali Deleu, Laurence Lins, Marie-Laure Fauconnier

**Affiliations:** 1 Laboratory of Chemistry of Natural Molecules, Gembloux Agro-Bio Tech, University of Liège, 5030 Gembloux, Belgium; m.genva@uliege.be (M.G.); kenne@gmx.com (T.K.K.); 2 Laboratory of Molecular Biophysics at Interfaces, Gembloux Agro-Bio Tech, University of Liège, 5030 Gembloux, Belgium; Magali.Deleu@uliege.be (M.D.); L.Lins@uliege.be (L.L.)

**Keywords:** olfaction, odor, sensory perception, flavor, volatile organic compounds, structure-function relationship, olfactory sense, odorant

## Abstract

The olfactory sense is the dominant sensory perception for many animals. When Richard Axel and Linda B. Buck received the Nobel Prize in 2004 for discovering the G protein-coupled receptors’ role in olfactory cells, they highlighted the importance of olfaction to the scientific community. Several theories have tried to explain how cells are able to distinguish such a wide variety of odorant molecules in a complex context in which enantiomers can result in completely different perceptions and structurally different molecules. Moreover, sex, age, cultural origin, and individual differences contribute to odor perception variations that complicate the picture. In this article, recent advances in olfaction theory are presented, and future trends in human olfaction such as structure-based odor prediction and artificial sniffing are discussed at the frontiers of chemistry, physiology, neurobiology, and machine learning.

## 1. Introduction

### 1.1. The Crucial Roles of the Olfactory Sense

It seems rational that the ability to recognize odor is common to every animal that has a nose, but this may become less obvious in single-cell micro-organisms. However, almost all organisms, including micro-organisms, can sense environmental cues through chemoreception [1]. The ability to perceive volatile organic compounds (VOCs) is found in various phyla from single-cell organisms to more complex ones such as mammals [2]. For most animals, the sense of smell is vital; the roles of odor perception can be related to various functions involved in guaranteeing survival and reproduction, such as finding food, avoiding danger, conspecific recognition, and searching for a mate [3,4,5,6]. Insects and certain vertebrate animals locate their prey through smell. To illustrate this, mosquitos locate mammals in order to suck their blood by detecting carbon dioxide, while common predators, especially felines, can recognize the scent of blood hundreds of miles away [7]. The sense of smell also allows animals to avoid danger by detecting foul-smelling food that may contain poisons and pathogens [8]. Finally, smell helps to build strong and positive ties between newborn animals, including humans, and their mothers. During pregnancy, women develop a particular pattern of volatile molecules which are useful to newborns to recognize their mother and to guide feeding immediately after birth and the first weeks of life [9].

### 1.2. Human Response to Odor

Smell is not simply a biological and psychological experience; it is also a social and cultural phenomenon [10], as the sense of smell differs depending on culture, age, gender, and health status [11]. Olfactory perception is widely influenced by background and semantic information, as two people with different cultural backgrounds can have different reactions when smelling the same thing. For example, Canadians can better describe the scent of maple than French people, the latter being better at describing lavender [12]. Odor recognition is also influenced by age, as children (<16 years old) and older people (>55 years old) are less sensitive to odor than young and middle-aged adults [13,14,15,16]. Variation in olfactory perception depending on sex is less obvious [13]. In a recent meta-analysis, it was shown that women generally outperform men in the identification and discrimination of odorants. Ladies usually also have lower threshold values for odorant molecules. Nevertheless, even if the differences are statistically significant, they are small [17].

### 1.3. Physical and Chemical Features of Odor

According to Richard Axel and Linda B. Buck (recipients of the 2004 Nobel Prize in medicine or physiology), smell is a chemical stimulus with a physiological response which can be due to one or a combination of odorant molecules [18]. The latter are volatilized chemical compounds, usually at small concentrations, that are perceived by animals, including humans, through the sense of olfaction [19]. The term “odor” is frequently used to name a scent that may or may not be pleasant, while the terms “fragrance” and “aroma” are mostly used by the cosmetic and food industries to describe a pleasant odor [19].

Odorant molecules include VOCs, which constitute a large class of low-molecular-weight (<300 Da) carbon-containing compounds characterized by their high vapor pressure (≥0.01 kPa at 20 °C) and high-to-moderate hydrophobicity [20]. An odor primarily originates from a compound volatilizing at ambient temperature and thus reaching the nose. However, the vapor pressure value is not sufficient to predict whether a compound is odorant or not. For example, humans, on the one hand, can smell NO_2_ but not CO_2_; on the other hand, they can smell relatively large molecules such as musk compounds [21,22]. Odorant molecules are not limited to carbon-containing compounds, as both organic and inorganic molecules may have a smell. For example, ammonia (NH_3_) is an inorganic compound that has a distinctive fishy scent [23]. Elemental chlorine gas (Cl_2_) has an acrid smell. Hydrogen sulfide (H_2_S) is another inorganic odorant with a rotten egg scent. It is widely accepted that humans are able to detect the presence of functional groups more easily with great reliability than a single elemental compound. This is the case of thiols (-SH), oxines (-NOH) and nitro groups (-NO_2_), respectively known for their sulfurous odor, green camphoraceous smell, and sweet ethereal character [23].

### 1.4. Odor and Structure Relationship

**Figure 1:**
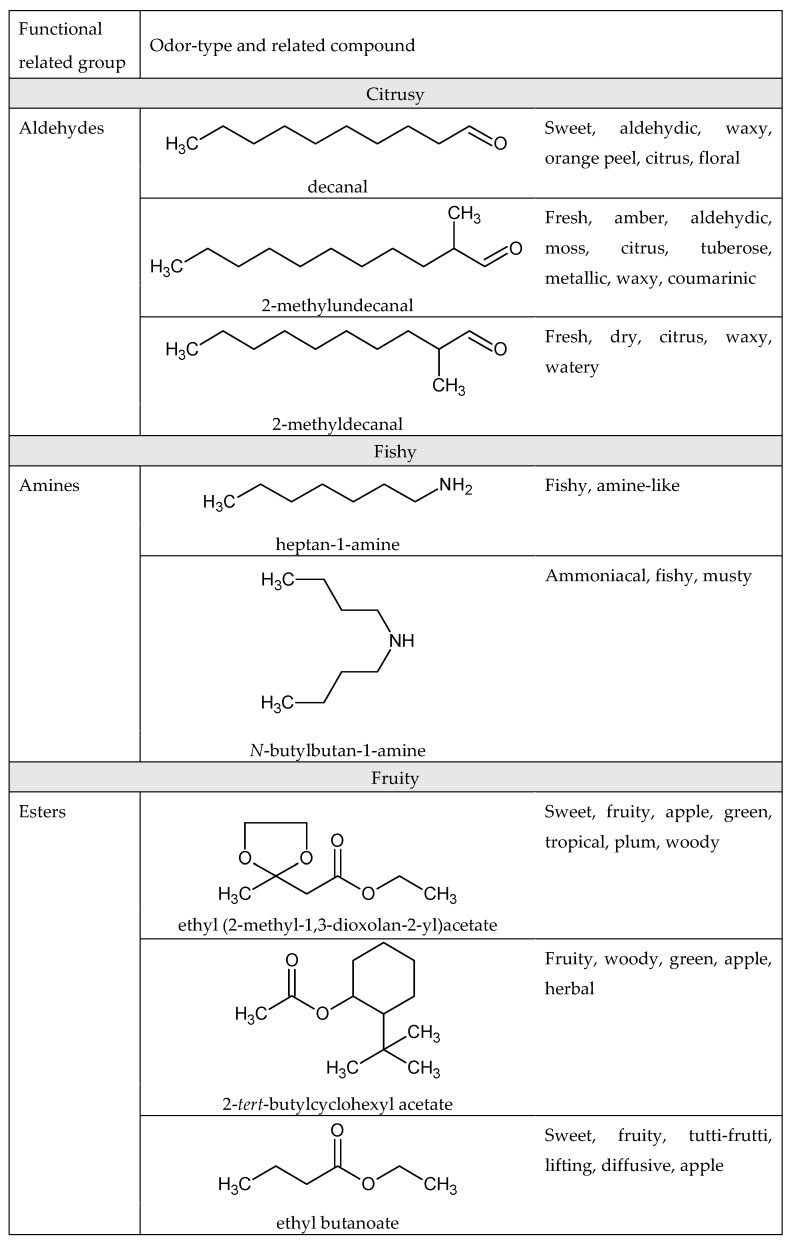
Examples of odorants with common functional groups and similar odors [23].

Odorant compounds that have the same functional group seem to have similar odors, as esters have a fruity and floral smell, lactones display a coconut or apricot character, amines have an animal/roasted scent, thiols have a rotten or alliaceous smell [24], volatile fatty acids have a sour to rancid smell, and aldehydes are associated with green odors such as grass cuttings or leaves [25,26]. Many studies have focused on the relationship between the molecular structure of a compound and its odor [27,28,29]. Indeed, resolving this issue could have a significant economic impact on perfume and aroma formulations. Some functional groups and their related smells are presented in Figure 1.

**Figure 2:**
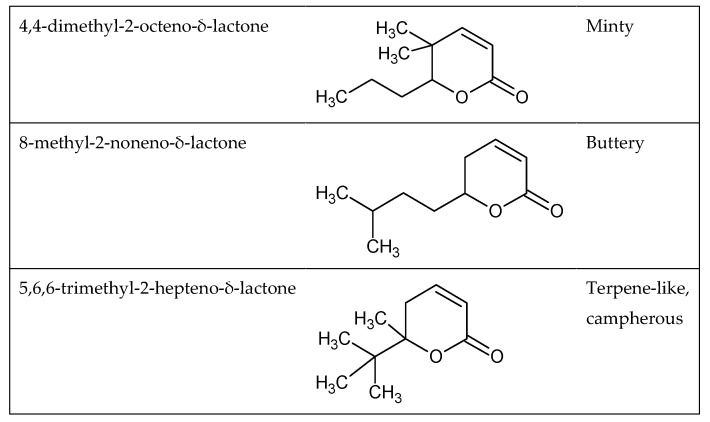
Examples of odorants with common functional groups and dissimilar odors [24].

However, only a few odors can be predicted based on the functional group of the molecule. Indeed, some odorant molecules have the same functional group but different odors. This is the case, for example, for 4,4-dimethyl-2-octeno-δ-lactone, 8-methyl-2-noneno-δ-lactone, and 5,6,6-trimethyl-2-hepteno-δ-lactone (Figure 2), which are three lactones that have very close structures but very distinctive smells. In fact, the first molecule has a minty odor, the second a buttery odor, and the last a terpene-like and camphorous character [30].

**Figure 3:**
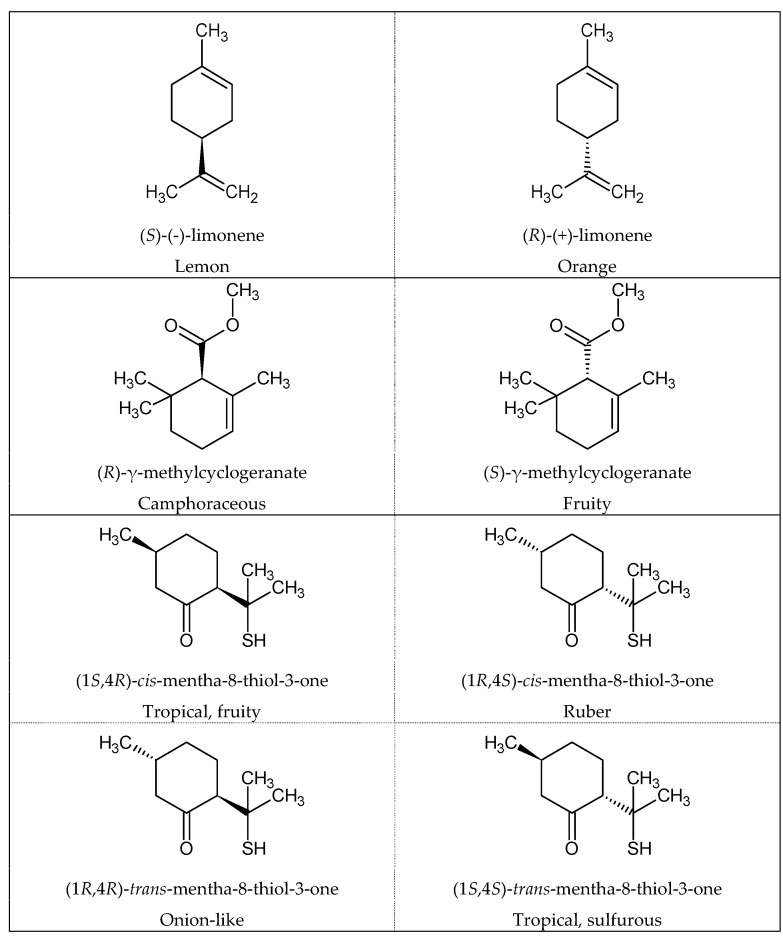
Examples of enantiomeric compounds with dissimilar odors [31,33].

Likewise, enantiomeric compounds, also known as optical isomers, obviously have the same chemical functions and are structurally close, but only as few as 5% of enantiomer couples have a similar smell [31]. A very common example used by many organic chemistry teachers is the two enantiomers of limonene: (*S*)-(-)-limonene smells like lemons, while (*R*)-(+)-limonene has the characteristic smell of orange. Another well-known example of an enantiomer couple with nonidentical smells is (*R*)-γ-methylcyclogeranate, which smells like camphor, and (*S*)-γ-methyl cyclogeranate, the scent of which is described as fruity [31]. When the molecule has two chiral centers, four different enantiomers exist, potentially leading to four different odors (e.g., mentha-8-thiol-3-ones) (Figure 3) [32].

**Figure 4:**
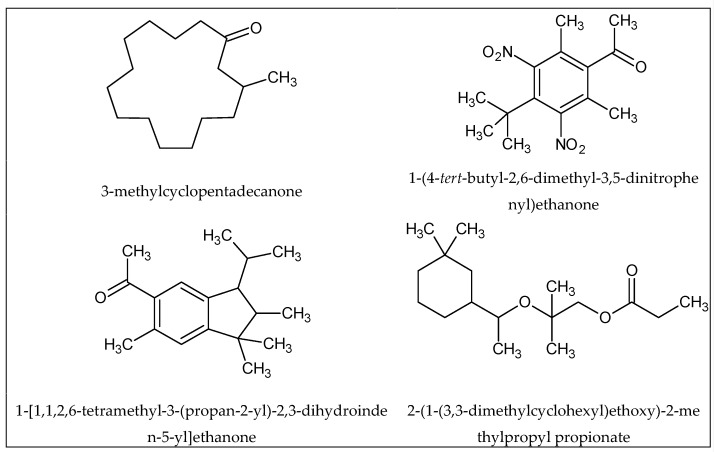
Examples of odorants with different structures and similar odors (musk) [32,37].

Finally, there is the case of structurally different organic compounds having a similar smell; for example, musk-related odors (Figure 4) [32,34,35,36,37].

**Figure 5:**
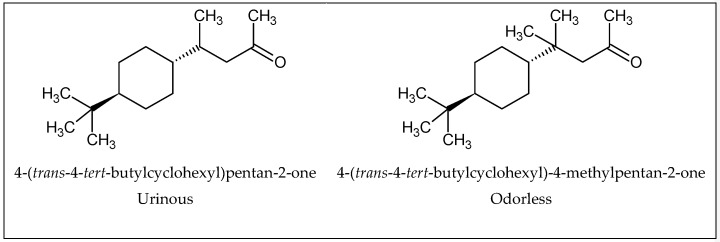
Example of the changing odorant character of a compound with slight structural modification.

Besides changing the odor, a small structural change to a molecule may cause a decrease in odor intensity. As an example (Figure 5), the left molecule has a urinous smell, while the molecule on the right, which only displays one extra CH_3_, is odorless [37,38].

A small change in the structure or functional group of a molecule can significantly alter its smell in a manner that a current prediction odor–structure model cannot completely explain.

## 2. Olfaction Mechanisms

### 2.1. The Physiology of Olfaction

**Figure 6:**
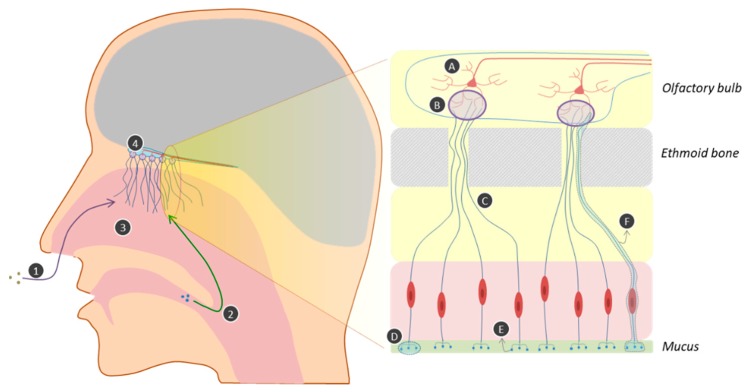
Mechanisms of human olfaction. **1.** Orthonasal olfaction; **2.** retronasal olfaction; **3.** nasal cavity; 4. olfactory bulb; **A.** mitral cell; **B.** glomerulus; C. axon; **D.** cilia; E. olfactory receptor; **F.** olfactory receptor neuron.

The olfactory system’s organization is remarkably similar in various animals, from insects to mammals, allowing the detection of a large array of structurally different molecules. The mechanism of olfaction can be divided into four main steps: airflow of the odorants, binding to receptors, transduction of the signal, and information processing [39]. Hereafter, we focus on human olfaction (Figure 6).

Odor molecules can reach the nasal cavity either through orthonasal olfaction (direct inhalation in front of the nose) or through the throat when the tongue pushes air to the back of the nasal cavity while chewing or drinking (retronasal olfaction) [33].

Inside the nasal cavity, odor perception is due to the interaction of volatile compounds with the olfactory receptor neurons (ORNs) that lie in the olfactory epithelium, which occupies a 3.7 cm^2^ zone in the upper part of the nasal cavity. The epithelium is covered by mucus and contains olfactory glands that secrete the enzymes found in the mucus [40]. Humans have around 12 million ORNs in each epithelium (right and left); as the olfaction system is bilateral, there are two of each structure [41].

Sensory transduction is the mechanism by which the information related to the odor detected by an ORN is transmitted to the brain. In fact, each ORN has at one end 20–30 cilia bathing in mucus and containing olfactory receptors (ORs) that bind to odor molecules to give an electrical response. The electrical message is transferred through the axon situated at the other end of the ORNs to nerve fibers situated at the back of the nasal cavity [33,40]. Each ORN expresses one receptor type in humans, whereas it was shown that a single neuron may express two different receptor types in other species [41]. The axons of ORNs pass through the ethmoid bone to form glomeruli. Glomeruli are composed of ORN axon terminals and the dendrites of mitral cells (second-order neurons). The axons of all the ORNs containing the same type of odor receptor are grouped in the same glomerulus. In humans, more than 5500 glomeruli are present, forming the olfactory bulb. The olfactory bulb is the place where the signal is treated and transmitted to the brain [41,42]. Axons of the mitral cells are grouped in olfactory tracts connected to the olfactory cortex. Until recently, the olfactory system was thought to be ipsilateral: the left epithelium is connected to the left bulb which itself is connected to the left cortex, but it is now known that the connectivity can also be contralateral [41]. Following olfactory stimuli, it has been shown that several brain zones are activated, such as the thalamus, amygdala, and orbitofrontal cortex, making olfaction a complex sensory experience [33].

### 2.2. Olfaction Theory: Studying Structure–Odor Relationships

The relationship between the molecular structure of a compound and its odor has been the subject of many studies. In this respect, two main schools of thought coexist: the global approach and the study of odorant molecule–receptor interaction. The global approach is quite descriptive and is ineffective at explaining the mechanisms underlying the olfaction chain. Nevertheless, these studies can be helpful for flavorists and perfumers in search of new raw materials for the products they develop [32,34,35,36,43,44,45,46]. The complete olfactory chain is complex and can lead to several biases. First of all, the way sniffing is performed, either rapidly or slowly, will influence the odor concentration reaching the nostril, but it seems that the olfactory bulb is able to distinguish odor concentration variation and nasal flow modification (mechanical perception) [41,47]. Retronasal and orthonasal olfaction can result in different perceptions, mainly due to the differential solubility of the odorants in the nasal or nasopharyngeal mucus, which differ in composition [48]. Prereceptor events, such as the enzymatic conversion of odorants in nasal mucus and binding to odorant-binding proteins, will also affect odor perception (e.g., conversion of aldehydes and esters to acids and alcohols) [49,50]. The nasal mucus is rich in odorant-binding proteins that have variable affinity for odorants depending on their chemical structure. The precise role of these odorant-binding proteins is quite hypothetical: trapping of highly concentrated odorants to protect the nasal epithelium, transporting hydrophobic compounds through the mucus, and removing odorants from the receptors to allow fast reactivation [51,52]. Post-receptor events can also complicate the understanding of the relationship between chemical structure and odor perception. Indeed, the transformation by the brain of the signal originating from the nose is not completely understood. The glomeruli are described as a temporally well-organized unit playing an important role in signal transformation that can be compared to the thalamus’ role in other sensory pathways; for example, helping to discriminate odor concentration changes for odor source localization [53,54,55,56]. Another problem emerges once the signal has been treated by the brain; that is, expressing the perceived odor in a way that everyone can understand. Unlike sight and hearing, which can be reduced to easily measurable physical parameters, olfaction is subjective, and personal characterization renders information sharing difficult even among professionals. Different reference scales such as the “field of odors” have been created using reference molecules to classify odors but are still far from a universal language for odor description [57,58].

Studies based on odorant–receptor interactions allow for a subtler comprehension of olfaction mechanisms but require extremely pure compounds, since a trace of a minor enantiomer can impair the quality of the conclusions. One must keep in mind that some odorants have a very low perception threshold; for example, the ethyl mercaptan added to propane as a warning agent can be detected at a concentration of 10^−7^ ng/L, the lowest one being pyrrolidino[1,2-e]-4H-2,4-dimethyl-1,3,5-dithiazine, the typical flavor of cooked shellfish, at 10^−8^ ng/L for the two isomers [32,41].

### 2.3. Odorant–Receptor Interactions

In 2004, Richard Axel and Linda B. Buck were awarded the Nobel Prize in medicine or physiology for their research on odorant receptors and the organization of the olfactory system [18]. These studies allowed a better understanding of the human smell mechanism through an elucidation of the olfactory system [18]. Before that, the sense of smell was less well understood among our senses, even though numerous different theories had been proposed [33]. Without going into deeper explanation, almost 30 theories [59,60] had been proposed regarding the olfactory sense before Richard Axel and Linda B. Buck’s discovery, including a model for the stimulation of the organ of smell based on the polarization effect [61], a model for the olfactory membrane [62], the piezoelectric effect [63], olfactory transduction based on the formation of a weak bound complex between an absorbed gas molecule and certain carotenoid pigments [64], an analogy with polar compounds in chromatographic systems [65], and a mechanism based on inelastic electron spectroscopy [59]. Among these theories, the stereochemical theory of olfaction, known as Amoore’s theory, had been very popular for quite some time [66]. Based on seven primary odors (e.g., camphor and flora), a general shape was associated with each primary odor. As an example, a camphoraceous odor was linked to a hydrophobic, ellipsoidal shape with a long axis of 0.95 nm and a short axis of 0.75 nm. This theory allowed the design of numerous commercially successful novel molecules such as Javanol®, Rossitol®, Belambre®, and Azurone®, but when the number of described specific anosmia (i.e., the inability to perceive specific odors) increased from 7, which was coherent with the model, to 90, the theory was discarded [66,67,68]. On the other hand, the vibrational theory considers that the detection of odorants is due to their vibrational frequencies and not to their shape; an electron transfer can occur between odorants and the active site of ORs. Several studies showing that humans are able to differentiate isotopomers (isomers with isotopic atoms) reinforced the theory because the latter differ in their molecular vibrational frequencies. However, in a recent study, Block et al. tested series of isotopomers of musk and other compounds for their capacity to activate specific odorant receptors in vitro and found no experimental evidence supporting the vibrational theory. Peri-receptor events or odorant contaminations, rather than vibrational effects at the receptor level, are suggested to explain the ability to discriminate isotopomers. It has since been proposed that the study of the nature of odorant–receptor recognition should be based on receptor activation mechanisms rather than odor perception [59].

**Figure 7:**
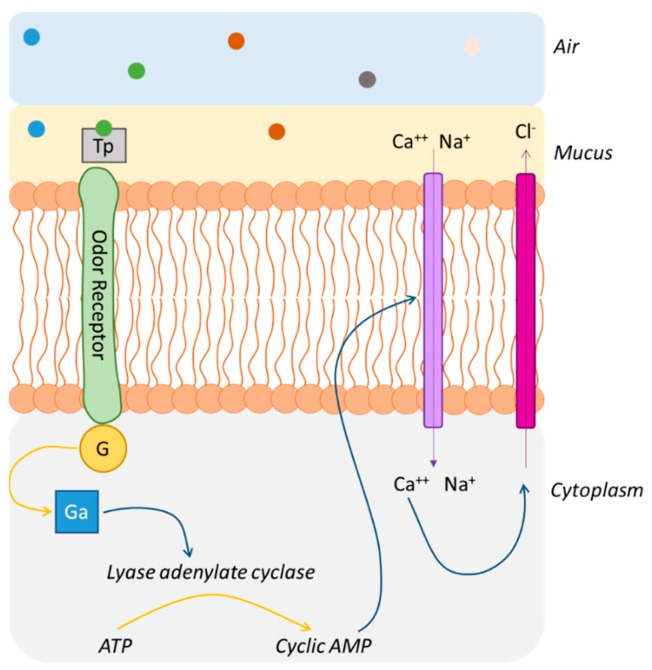
Cascade of reactions occurring when an odorant molecule enters the nasal cavity. Odorants cross the mucus directly or via transport proteins (Tp) and then reach the odor receptor, which undergoes structural modifications and activates G proteins (G). An active subunit (Ga) is liberated, activating the lyase adenylate cyclase, resulting in the conversion of adenosine triphosphate (ATP) into cyclic adenosine monophosphate (cAMP). The cAMP is able to open cyclic nucleotide-gated ion channels, allowing Ca^2+^ and Na^+^ ions to enter the cell, which results in the depolarization of the odor receptor neuron (ORN) and transmission of the information to the brain.

Buck and Axel hypothesized that the binding of odor molecules to specific surface receptors (GTP-binding protein-coupled receptors, or GPCR) activates specific G proteins. GPCRs are transmembrane proteins that cross the membrane seven times with an extracellular N-terminus and an intracellular C-terminus (seven α-helices and six loops) [18,33]. Because the direct identification of those transmembrane proteins is difficult in biological samples, 18 different members of a large multigene family only expressed in the olfactory epithelium were cloned and characterized [18]. Genetic studies have shown that OR proteins are mainly expressed in ORNs and are highly variable, especially in the transmembrane domains—3, 4, and 5, presumably—where the odorant binds the protein [18]. Figure 7 presents the cascade of reactions occurring when an odorant molecule enters the nasal cavity. The odorant crosses the mucus directly or via transport proteins and then reaches the odor receptor. After the binding of the odorant, the receptor undergoes structural modifications and activates G proteins that are located on the cytoplasmic side. An active subunit called Ga is liberated, activating, in turn, the lyase adenylate cyclase, resulting in the conversion of adenosine triphosphate (ATP) into cyclic adenosine monophosphate (cAMP). The cAMP is able to open cyclic nucleotide-gated ion channels allowing Ca^2+^ and Na^+^ ions to enter the cell, which results in the depolarization of the ORN and transmission of the information to the brain. Nevertheless, the entry of Ca^++^ ions opens Ca^++^-dependent Cl^-^ channels, which increases the depolarization [18,33,69,70].

The number of odorant molecules that humans are able to distinguish is huge, but their exact number is still controversial, with estimates ranging from 400,000 to 1 million [2]. Two theories could explain this ability: a large number of olfactory receptors, each interacting with a few odorants, or a small number of olfactory receptors responding to a great number of odorants in a combinatorial way. With only 396 unique olfactory receptors [71], only the second hypothesis is plausible, which is considerably different than the classical “lock and key” mechanism [2]. Compared with other mammals such as rodents, humans have fewer olfactory receptors (1130 in mice) [71] and smaller olfactory bulbs, but the number of neurons they contain is comparable [72].

**Figure 8:**
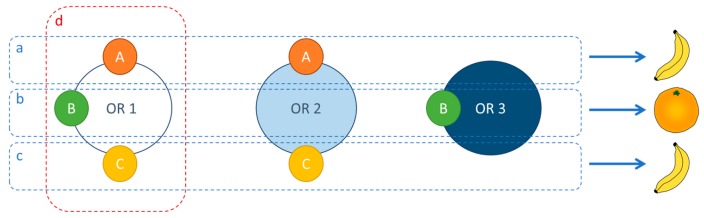
Olfactory receptor (OR) responses to odorants. Humans have approximatively 396 olfactory receptors (OR 1, OR 2, OR 3, etc.). A single olfactory receptor is able to recognize different odorant molecules. As an example, OR 1 is able to recognize molecules A, B, and C (d). The identification of a particular odorant is caused by the activation of a group of receptors with a special pattern (a, b, and c). For example, odorant A is recognized by OR 1 and OR 2 as a banana flavor (**a**). Molecule B is, in turn, recognized by OR 1 and OR 3 and identified as an orange flavor (**b**). Also, two distinct molecules can be recognized by the same receptors and identified as having the same odor (a and c). Indeed, odorant C is also recognized by OR 1 and OR 2 as a banana flavor (**c**).

Rather than binding specific ligands, olfactory receptors have affinity for a range of odorants, and an odorant molecule can bind several receptors with varying affinities depending on its physicochemical properties, such as the molecular volume. Likewise, the identification of a particular odorant is not due to the activation of one single receptor but to the activation of a group of receptors with a special pattern which is typical for combinatorial coding. It has been shown that receptors can be broadly tuned and respond to many different odorants, being most responsive to structurally similar odorants, or narrowly tuned, responding to a small group of odorants, as depicted in Figure 8 [23].

As a complement, higher concentrations of odorants activate a greater number of receptors. Hence, the number of activated receptors is linked both to the identity of an odorant as well as its concentration [39]. Another concern is temporality. An odorant can cause a long-lasting response in some olfactory receptors and a shorter response in others. As a corollary, an individual olfactory receptor can give a short answer to some molecules and a long one to other compounds [39]. Furthermore, the activation of olfactory receptors is not the only regulation pathway in olfaction, as some olfactory receptors can also be inhibited by some odorants [39]. Since a given ORN expresses only one class of odorant receptor in humans, and as neurons containing the same type of receptor send their axons to one or a few glomeruli of the olfactory bulb, the study of the interaction between a receptor and an odorant molecule is a key point in olfaction research [73].

Sequences of thousands of receptors have been discovered from genomes. ORs, representing more than 2% of the human genome and 4% of our proteome, belong to the typical class A GPCR motifs. Nevertheless, ORs show a low sequence identity with nonolfactory proteins of this class, as OR conserved motifs are either dissimilar to or more complex than those present in sequences of other class A GPCR. Since no 3D structure of ORs either in the active or inactive state has been resolved to date, molecular modeling approaches (homology modeling, docking, and molecular dynamics, notably) coupled to sequence conservation and site-directed mutagenesis have helped to predict 3D models of ORs and their binding to diverse ligands. The binding pocket is notably composed of hydrophobic transmembrane (TM) residues from TM3, 5, 6, and 7 [74,75,76,77,78], such as Phe 104, that could contribute to stabilizing the ligand through an interaction between the aromatic cycle and double bonds from odorant molecules [74]. The activation mechanisms are still poorly understood since the highly conserved motifs, critical for the activation of nonolfactory GPCRs, are not present. Some of these mechanisms’ aspects have been recently elucidated by using molecular dynamics and point mutations, involving the highly conserved Y252 in helix 6 and identifying the ionic lock residues between TM3 and TM6, which usually induce conformational change and subsequent activation [74]. Even though these models have substantially advanced our understanding of the recognition and activation of ORs, the structural elucidation of olfactory GPCRs would provide a great advance in the field of olfaction, as was in the case of rhodopsin, the first G-coupled receptor to be crystallized, which helped us to better understand light perception [39].

In mammals, olfactory receptors are not restricted to odorant receptors of the main olfactory epithelium. In mice, a commonly used model, the olfactory system contains different chemosensory subsystems counting the following olfactory receptors: odorant receptors, vomeronasal receptors, trace amine-associated receptors, formyl peptide receptors, and guanylyl cyclase receptors, most of which belong to the GPCR class. The relationship between the main olfactory system and accessory olfactory systems is complex and needs further investigation, especially for species other than mouse [2,23,79]. The vomeronasal organ is also present in insects, where it allows, along with antennas, the perception of pheromones, for example. Those molecules are semiochemicals known to influence communication in all living organisms [80]. According to their function, pheromones play key roles in animal alarm, releaser, territorial information, and sex [3,81]. In humans, the vomeronasal organ is vestigial and nonfunctional [81], and the existence of functional accessory olfactory systems is still unclear [41]. Furthermore, olfactory receptors have been described in nontypical olfactory tissues such as myocardial and erythroid cells, ganglia, spleen, colon, prostate, and testis [82]. Olfactory receptors were found in mature sperm, revealing a potential role in sperm–oocyte chemiotaxis [82].

## 3. New Trends in Olfaction Studies

### 3.1. Importance and Diversity of Human Olfaction

Since the 19th century and Broca’s claim that humans are “nonsmellers”, the belief that humans have an underdeveloped sense of smell compared with other mammals is deeply rooted in the scientific community [72]. As a matter of fact, the American Medical Association has stated that loss of vision and audition resulted, respectively, in a disability rate of 85% and 35%, whereas it was only 3% for olfaction loss [41]. Recent studies contradict this 19th century myth, revealing that humans have both very good capacities for detecting and discriminating different odors [72,83]. The importance of odors and their influence on mood, cognition, and behavior is now recognized [84].

Differences between individuals in perceiving odorants have received more attention recently. Human olfaction perception is extremely variable for both specific sensitivity and general olfactory acuity, with sensitivity varying several orders of magnitude between individuals [85]. The polymorphism of olfactory receptors constitutes the molecular basis of such individual differences [85]. Specific anosmia, specific hyposmia (reduced ability to detect an odor), and the less-studied specific hyperosmia (increased olfactory acuity) occurrences are extremely numerous, which are, from a statistical point of view, more the rule than the exception [85,86]. The first logical explanation of such large olfactory perception diversity is genetic. Among the olfactory receptor genes and pseudogenes of the human genome, more than 60% present damaging single-nucleotide polymorphisms (SNPs) in their coding sequence. SNPs are a major source of human genetic variability and, when they are present in a coding sequence of the genome, they cause segregation between intact and disrupted alleles in the population. Even though a significant concordance can be observed for several odorant thresholds, it is suggested that olfactory acuity is a complex trait due to olfactory receptor variations as well as differences in the downstream treatment of the information in the signaling pathway [85,87]. The complex origin of anosmia, which is not only due to olfactory receptor genetic variations, is supported by the observation that training can be used to improve sensitivity towards specific odorants which were initially undetectable [86]. The plasticity of the olfactory system in adults has been shown by successfully training volunteers to discriminate between odorant enantiomers [88].

Olfaction studies are no longer restricted to perfumers and flavorists, as illustrated by military interest in the field [89]. The importance of the olfactory sense is now gaining the attention of the medical domain because an impaired olfactory system impacts physical health, nutrition and eating pleasure, and more generally, the quality of life [84,90]. Each of the four steps of the olfaction process can be affected: nasal airflow can be impacted by nasal polyposis, chronic rhinitis can result in peri-receptor event alteration, transduction via the primary olfactory neurons can be impaired by viral rhinitis, and neurodegenerative diseases are mainly responsible for brain processing problems [40,90]. Raising awareness of olfaction’s importance in human health is crucial. Smell loss can be an early sign of neurodegenerative diseases such as Alzheimer’s disease or Parkinson’s disease and can assist in their diagnosis [90].

### 3.2. Electronic Nose and Machine Learning

High interindividual variations in olfaction capacities and increasing sets of data pave the way for new fields in olfaction studies, such as the electronic nose and machine learning.

Electronic noses are not new; the first one was designed four decades ago and consisted of simple gas detectors, the ratio of the signals from the different transducers being processed to identify an odor [91]. Electronic noses are made up of an array of different gas sensors that can interact with volatile molecules and produce a signal, such as an electrical or optical signal, that can be amplified and finally processed [92]. Progress in electronic nose technology is due to concomitant improvements of sensor properties, signal preparation and pattern recognition, as reviewed in [93]. To cope with the high sensitivity, high selectivity, and the number of odorants potentially detected by the human nose, numerous studies have already been undertaken, but many more must still be performed before designing an instrument similar to the human olfactory system that can work in various environmental conditions [94]. It remains true that some volatile molecules can be detected by artificial olfaction machines but not by the human nose. The diversity of detected odors can be improved by increasing the number of specific sensing elements, even though appropriate sensitivity is difficult to reach, as human nose detection limits are at least 3–5 orders of magnitude lower than those of actual gas detectors [92]. The selectivity is linked to the type of sensing element used. Metal oxides and conducting polymers have poor selectivity [92], while semiconducting oxide nanobelts are more stable and highly sensitive [95]. Chiral polymer composite allows for the differential detection of enantiomeric volatile compounds [96]. More recently, it was proposed to use olfactory receptor proteins as sensing elements, but as they are transmembrane proteins, it was impossible to obtain the required stability. Odorant-binding proteins are more appropriate candidates as they are soluble and extremely stable with temperature and in organic solvents [94]. The main problems that remain to be solved are the need for an aqueous interface and the low dissociation kinetics which are incompatible with continuous monitoring of odorants. Design of site-specific mutated proteins with modified ligand-binding specificity could be a solution [94]. Although an instrument capable of discriminating odors with human-like performance still needs to be designed, the application domains are increasing with technological improvements in the military field [89], environmental monitoring [97], medical diagnostics [98], and food and beverage quality control [99,100,101].

Questions still remain regarding the multifaceted olfactory code and the complex and high-dimensional aspects of data from olfactory research. During the last two decades, artificial neural networks were used to help elucidate structure–odor relationships: prediction of the camphoraceous or fruity odor of aliphatic alcohols [102], the musk odor of tetralin and indan compounds [103], the aroma quality and threshold values of pyrazines [104], and so forth. More recently, machine learning was used to facilitate complex data-driven research in the olfactory field; for example, to predict the response of olfactory receptors [105]. Machine learning methods are increasingly widely used in human olfactory research at different levels: physiology of odor detection and recognition, olfactory acuity as diagnostic biomarkers, and odor recognition from physicochemical properties of volatiles (for review, see [106]).

## 4. Conclusions

Determining whether it is possible to predict the odor of a molecule based on its structure is certainly not trivial. The literature is replete with descriptions of odor and threshold values for thousands of natural and synthetic odorants, which can be useful for anyone wishing to know what smell is associated with an already-known molecule. Structural similarity can be used in some cases to predict the odor of a particular compound but, as mentioned before, it is far from being 100% reliable.

Recent progress in chemistry, physiology, neurobiology, and genetics with the help of bioinformatics and machine learning should allow the better understanding of the human olfaction system. Studying olfactory receptor–odorant interactions and the activation mechanisms is certainly the most attractive approach in order to predict an odor based on molecular structure. Nowadays, it seems impossible to test every single olfactory receptor with every possible odorant, but with the help of bioinformatics approaches, this goal is perhaps reachable, especially if the 3D structure of ORs were found. The combinatorial coding scheme of odorants requires a better understanding of how the combination of OR activation is processed in the brain to link odor to olfactory perception. Taking into account the enormous datasets generated by combinatorial coding, machine learning can be helpful. Intrinsic signal imaging of the olfactory bulb has been suggested by some authors, but technical improvements are still required. Depending on the goal of each study, a global approach may be appropriate. For example, when the main question is not knowing how the olfactory system works but rather whether the consumer will enjoy the food/perfume for commercial applications, trained panelists would be the best solution to provide sensory evaluations of the products and to reflect individual variability [107,108].

In many traditions, sniffing food is considered impolite. This cultural issue has gradually contributed to the decline of olfaction in everyday life. From a public health point of view, it is crucial to restore the pivotal role of olfaction among our five senses by changing mentalities and encouraging children to smell and enjoy what they eat. Elders should also stimulate their olfaction to counteract their loss in olfactory ability, since this is linked to a loss of appetite, which can have dramatic consequences on health and even be a marker of neurodegenerative disorders.
